# Comparison of Sofia* Legionella* FIA and BinaxNOW®* Legionella* urinary antigen card in two national reference centers

**DOI:** 10.1007/s10096-015-2415-9

**Published:** 2015-06-20

**Authors:** L. Beraud, K. Gervasoni, A. M. Freydiere, G. Descours, A. G. Ranc, F. Vandenesch, G. Lina, V. Gaia, S. Jarraud

**Affiliations:** French Reference Center for Legionella, Laboratory of Microbiology, Groupe Hospitalier Est, Hospices Civils de Lyon, Lyon, France; CIRI, International Center for Infectiology Research, Inserm, U1111 ; CNRS ; UMR5308, Université Lyon 1 ; École Normale Supérieure de Lyon, Lyon, F-69008 France; Reference Center for Legionella, Laboratory of Microbiology, Department of Laboratory Medecine, Ente Ospedaliero Cantonale, Bellinzona, Switzerland

## Abstract

The Sofia Legionella Fluorescence Immunoassay (FIA; Quidel) is a recently introduced rapid immunochromatographic diagnostic test for Legionnaires’ disease using immunofluorescence technology designed to enhance its sensitivity. The aim of this study was to evaluate its performance for the detection of urinary antigens for* Legionella pneumophila* serogroup 1 in two National Reference Centers for* Legionella*. The sensitivity and specificity of the Sofia* Legionella* FIA test were determined in concentrated and nonconcentrated urine samples, before and after boiling, in comparison with the BinaxNOW®* Legionella* Urinary Antigen Card (UAC; Alere). Compared with BinaxNOW®* Legionella* UAC, the sensitivity of the Sofia* Legionella* test was slightly higher in nonconcentrated urine samples and was identical in concentrated urine samples. The specificity of the Sofia* Legionella* FIA test was highly reduced by the concentration of urine samples. In nonconcentrated samples, a lack of specificity was observed in 2.3 % of samples, all of them resolved by heat treatment. The Sofia* Legionella* FIA is a sensitive test for detecting* Legionella* urinary antigens with no previous urine concentration. However, all positive samples have to be re-tested after boiling to reach a high specificity. The reading is automatized on the Sofia analyzer, which can be connected to laboratory information systems, facilitating accurate and rapid reporting of results.

## Introduction

The accurate diagnosis of *Legionella pneumonia* is important for the treatment of patients by health care providers and for the control of *Legionella* epidemics by public health officials. Rapid urinary antigen detection kits such as lateral flow immunochromatographic assays detecting *L. pneumophila* serogroup 1 are the first-line diagnostic tests for Legionnaires’ disease. In Europe, the 80 % of cases were diagnosed using urinary antigen kits in 2009–2010 [1].

Various immunochromatographic membrane kits requiring no specialized laboratory equipment are commercially available. These tests are easy to perform and provide results in a few minutes, but they have not demonstrated optimal sensitivity [2–4]. Two approaches have been proposed to increase the sensitivity of the commercialized kits that are available: additional readings after a longer incubation time [2, 4–8] and the concentration of urine samples by centrifugation using filter units. Several studies showed an increase in the sensitivity of the BinaxNOW® *Legionella* Urinary Antigen Card (UAC) by using concentrated urine samples [4, 9–11].

The Sofia *Legionella* Fluorescence Immunoassay (FIA; Quidel, San Diego, CA, USA) is a recently introduced rapid diagnostic test for Legionnaires’ disease (LD) that uses immunofluorescence technology coupled with an automatic reader to enhance its sensitivity. To date, no study has been published on the performance of this test compared with the BinaxNOW® *Legionella* UAC (Alere, Jouy-en-Josas, France), the kit that is most commonly used. The aim of this study was to evaluate the performance of Sofia *Legionella* FIA in comparison with BinaxNOW® *Legionella* UAC in concentrated and nonconcentrated urine samples at two National Reference Centers for *Legionella*.

## Materials and methods

### Clinical specimens

A total of 269 urine samples were tested in the French and Swiss National Reference Centers (centers 1 and 2 respectively: 199 in center 1 and 70 in center 2), corresponding to 179 prospective samples submitted for *Legionella* urinary antigen detection between January and March 2013 (150 in center 1 and 29 in center 2) and 90 repository urine samples collected from 2009 to 2013 from patients with known LD and stored at −20 °C (49 in center 1 and 41 in center 2). Previously, urine samples had been classified as positive based on a BinaxNOW®* Legionella* UAC result obtained from concentrated urine samples.

A case of LD was defined according to the European Legionnaires’ Disease Surveillance Network (ELDSNet) criteria [12], i.e., clinical and/or radiological evidence of pneumonia associated with positive urinary antigens and/or positive culture or PCR on respiratory samples. In our study, cases were diagnosed by positive urinary antigens using BinaxNOW® *Legionella* UAC on concentrated urine samples. Twenty-five cases were confirmed by culture on respiratory samples with *Legionella pneumophila* serogroup 1 strain and 7 of them were Mab3/1-negative.

### Legionella urinary antigen detection

The BinaxNOW® *Legionella* UAC is a colorimetric immunochromatographic test. A sample was considered positive when both the control line and the test line were visible after 15 min of incubation at room temperature.

The Sofia *Legionella* FIA kit employs a lateral-flow immunofluorescence technique. The signal is only visible under UV light. The reading is performed after 10 min of incubation and interpreted using the SOFIA^TM^ analyzer, which automatically scans the test strip, collects and analyzes the fluorescence data, and then calculates and reports the result in about 1 min.

With the Binax *Legionella* Urinary Antigen EIA kit (Alere), a sample was considered positive when the mean absorbance value was three times the mean value of the negative control.

### Method

The tests were performed according to the manufacturers’ instructions. In addition, some samples were concentrated and heat-treated before testing. Urine samples yielding discrepant results before and after heat treatment were checked by using the Binax *Legionella* Urinary Antigen EIA kit.

Nonconcentrated urine samples were tested using Sofia *Legionella* FIA and the results were compared with those of nonconcentrated (centers 1 and 2) and those of concentrated urine samples (center 1 only) tested simultaneously using BinaxNOW® AUC.

Urinary concentration was performed in center 1 by centrifugation at 4,000 *g* for 10 min using Amikon Ultra-4 Ultracel-10 k (Millipore Corporation, Bedford, MS, USA) [13].

In the two centers, urine samples yielding a positive result were retested after heating at 100 °C for 5 min and centrifugation for 15 min at 1,000 *g* to exclude false-positive results [14]. Any sample yielding a positive result after heating was considered true-positive. Any urine sample positive before heating and negative after heating was considered false-positive.

## Results

Of the 269 nonconcentrated urine samples tested, the Sofia *Legionella* FIA test and the BinaxNOW® *Legionella* UAC yielded 172 and 181 negative results and 97 and 88 positive results respectively (Table 1). Of the 9 discrepant samples yielding a positive result with the Sofia *Legionella* FIA test, but a negative one with the BinaxNOW® *Legionella* UAC, 5 urine samples (4 in center 1 and 1 in center 2) yielded a negative result with the Sofia *Legionella* FIA test after heating. Four of them with a sufficient amount to be checked with the BinaxNOW® EIA showed a confirmed negative result. No respiratory samples were available for these 5 patients. The 4 other urine samples (2 in center 1 and 2 in center 2) remained positive with the Sofia *Legionella* FIA test after heating and yielded a positive result with the BinaxNOW® EIA before and after heating. For 2 of these patients, a *Legionella* PCR performed on respiratory samples was positive and in addition, for one of them a *L. pneumophila* serogroup 1-PCR was also positive. For the other one, the *L. pneumophila* serogroup 1-PCR was negative.

**Table 1:** Comparison of the results of the Sofia *Legionella* fluorescent immunoassay (FIA) and the BinaxNOW® *Legionella* urinary antigen card (UAC) using nonconcentrated urine samples (*n* = 269)

		BinaxNOW® *Legionella* UAC nonconcentrated urine				
		−		+		Total
		Center 1	Center 2	Center 1	Center 2	
Sofia *Legionella* FIA Nonconcentrated urine	− +	146	26	0	0	172
		6^a^	3^a^	47	41	97
	Total	181		88		269

^a^Of the 9 discrepant results with nonconcentrated urine samples, 5 (4 in center 1 and 1 in center 2) yielded a negative result after heating and corresponded to a false-positive diagnosis using the Sofia *Legionella* FIA test without heating. The 4 other urine samples (2 in center 1 and 2 in center 2) remained positive after heating and corresponded to true-positive diagnosis

As several studies demonstrated that urine sample concentration increased the sensitivity of urinary tests, the results of the BinaxNOW® *Legionella* UAC on concentrated urine samples were compared with those of the Sofia *Legionella* FIA on nonconcentrated urine samples in center 1 (*n* = 199; Table 2). The two reagents detected 49 positive samples, confirming the presence of *Legionella* urinary antigen. Among them, 2 samples had not been detected by the BinaxNOW® *Legionella* UAC before concentration. Four discrepant results were observed, yielding a positive result with the Sofia *Legionella* FIA and a negative one with the BinaxNOW® *Legionella* UAC. These samples were confirmed as negative by the Sofia *Legionella* FIA after heating, corresponding to false-positive results as previously described in Table 1.

**Table 2:** Comparison of the results of the Sofia *Legionella* fluorescent immunoassay (FIA) using nonconcentrated urine samples and the BinaxNOW® *Legionella* urinary antigen card (UAC) using concentrated urine samples in center 1 (*n* = 199)

		BinaxNOW® *Legionella* UAC concentrated urine		
		−	+	Total
Sofia *Legionella* FIA Nonconcentrated urine	− +	146	0	146
		4^a^	49	53
	Total	150	49	199

^a^These 4 urine samples yielded a negative result after heating and corresponded to the same samples as in the cells marked ^a^ in Table 1 (false-positive results)

Although the manufacturer of the Sofia *Legionella* FIA does not recommend urine concentration, we evaluated its impact on 199 concentrated urine samples in center 1. Eleven urine samples were detected as being positive with use of the Sofia *Legionella* FIA, but negative with use of the BinaxNOW® *Legionella* UAC. Among these samples, 9 turned out to have negative results after heating, confirming false positivity. In 1 case there was not enough of the urine sample available for retesting after heating; thus, it was classified as invalid. The last sample remained positive after heating, but was detected as being negative by the BinaxNOW® EIA. *Legionella* spp. and *L. pneumophila* PCR performed on respiratory samples were negative for this patient.

## Discussion

In this work, we evaluated the Sofia *Legionella* FIA, a recently introduced rapid diagnostic test for the detection of *L. pneumophila* serogroup 1 urinary antigens, which uses immunofluorescence technology to enhance its sensitivity.

Although the manufacturers do not recommend urine concentration, the centrifugal ultrafiltration method for rapid concentration of *L. pneumophila* urinary antigen was evaluated. The *Legionella* urinary antigen is stable at 100 °C for 30 min [15]; therefore, all positive samples were retested as described previously after boiling to suppress nonspecific reactions and confirm positive results [9, 14, 16, 17].

Our results demonstrate that the Sofia *Legionella* FIA test exhibits higher sensitivity than the BinaxNOW® *Legionella* UAC, since it allowed the detection of all the LD-positive patients with nonconcentrated urine samples. This study also confirms that the sensitivity of BinaxNOW® *Legionella* UAC is enhanced by the concentration of urine without any decrease in specificity. As a matter of fact, 2 nonconcentrated urine samples that tested negative with the BinaxNOW® *Legionella* UAC yielded a positive result after concentration. Thus, these two tests demonstrated similar performance in terms of sensitivity only if urine samples are concentrated for the BinaxNOW® *Legionella* UAC.

The major limitation of the Sofia *Legionella* FIA is the lower specificity compared with the BinaxNOW® *Legionella* UAC that was detected in both concentrated and nonconcentrated urine samples. As a matter of fact, Quidel recommends using the Sofia *Legionella* FIA in nonconcentrated urine samples. Our results confirm this important point, since the number of false-positive results increases from 4 before to 11 after concentration. Moreover, 1 out of the 11 false-positive results in concentrated urine samples remained positive after heating.

These results of sensitivity and specificity were further confirmed in 214 new prospective urine samples tested between March and June 2014 in center 1 with Sofia *Legionella* FIA performed on nonconcentrated samples and the BinaxNOW® *Legionella* UAC on concentrated samples. Three positive urine samples were equally detected by the two kits and boiling urine samples eliminated 6 false-positive results with use of the Sofia *Legionella* FIA.

Finally, without heat treatment, a lack of specificity was observed for 2.3 % of all samples tested in this study (11 out of 483). Because of the consequences of false-positive results on the patient outcome and on outbreak investigation [17, 18], the recommendation of boiling any positive urine to differentiate a false-positive result from a true one should be recommended in the manufacturer’s instructions.

The Sofia *Legionella* FIA may represent a next step in the evolution of immunoassays, combining lateral flow and fluorescent antibody detection formats. The choice of fluorescent rather than colorimetric detection is at least partially responsible for the improved sensitivity demonstrated by the Sofia *Legionella* FIA, and this improvement has also been demonstrated for *Influenza* virus detection in comparison with several colorimetric lateral flow devices [19–22]. Because of its automated reading rather than subjective readings of the colorimetric bands, this assay produces objective data. Moreover, the Sofia analyzer can be connected to laboratory information systems to facilitate the accurate and rapid reporting of results.
